# Exploring the Influence of Tropical Cyclones on Regional Air Quality Using Multimodal Deep Learning Techniques

**DOI:** 10.3390/s24216983

**Published:** 2024-10-30

**Authors:** Muhammad Waqar Younis, Saritha, Bhavya Kallapu, Rama Moorthy Hejamadi, Jeny Jijo, Raghunandan Kemmannu Ramesh, Muhammad Aslam, Syeda Fizzah Jilani

**Affiliations:** 1 Department of Computer Science, Aberystwyth University, Penglais, Aberystwyth SY23 3DB, UK; mwy1@aber.ac.uk (M.W.Y.); mua19@aber.ac.uk (M.A.); 2 Department of Computer Science and Engineering, PES University, Bangalore 560085, Karnataka, India; jennyjijo@pes.edu; 3 Department of Mathematics, NMAM Institute of Technology, Nitte (Deemed to Be University), Mangalore 575018, Karnataka, India; bhavyak@nitte.edu.in; 4 Department of Computer Applications, Nitte Institute of Professional Education, Nitte (Deemed to Be University), Mangalore 575018, Karnataka, India; ramamoorthy.h@nitte.edu.in; 5 Department of Computer Science and Engineering, NMAM Institute of Technology, Nitte (Deemed to Be University), Mangalore 575018, Karnataka, India; 6 Department of Physics, Physical Sciences Building, Aberystwyth University, Aberystwyth SY23 3BZ, UK; sfj7@aber.ac.uk

**Keywords:** tropical cyclones, air quality, multimodal framework, ConvLSTM (Convolutional Long Short-Term Memory), GAN (Generative Adversarial Network), CNN (Convolutional Neural Network)

## Abstract

Tropical cyclones (TC) are dynamic atmospheric phenomena featuring extreme low-pressure systems and powerful winds, known for their devastating impacts on weather and the environment. The main purpose of this paper is to consider the subtle involvement of TCs in the air quality index (AQI), focusing on aspects related to the air quality before, during and after cyclones. This research employs multimodal methods, which include meteorological data and different satellite observations. Deep learning approaches, i.e., ConvLSTM, CNN and Real-ESRGAN models, are combined with a regression model to analyze the temporal variability in the air quality associated with tropical cyclones. Deep learning models are deployed to uncover complex patterns and non-linear interdependencies between cyclones’ features and the AQI to give predictive insights into the air quality fluctuations throughout the different stages of tropical cyclones. Furthermore, this study explores the aftermaths of TCs in terms of the air quality with respect to post-cyclone recovery. The findings offer an enhanced view of the role of TCs in the regional or global air quality, which will be useful for policymakers, meteorologists and environmental researchers. Utilizing a CNN for tropical cyclone (TC) classification and the extra trees regressor (ETR) for AQI prediction results in accuracy of 92.02% for the CNN and an $R^{2}$ of 83.33% for the ETR. Hence, this work adds to our knowledge and enlightens us on the complex interactions between TCs and the air quality, highlighting wider public health concerns regarding climate adaptation and urban renewal.

## 1. Introduction

Over the past fifty years, 1942 TC-related disasters have occurred, and they have claimed the lives of more than 779,324 people, with economic losses of more than USD 14 trillion. Around the world, about 85 tropical storms develop over the warm tropical oceans and extend from 25° S to 25° N each year. Among these storms, about forty-five transform into hurricanes or typhoons [1]. A tropical cyclone is an area of disturbed weather characterized by a concentrated swirl of winds that move in a circular manner around the center of a massive and low-pressure air mass [2]. This is a major meteorological event characterized by large fluctuations in the temperature, wind speed and pressure. This occurs as a warm core characterized by circular wind moving around a specific center [3]; it rotates anticlockwise in the north and clockwise in the south. It is the vertical temperature difference that provides the impetus for tropical cyclones. Storm surges are one of the most severe consequences associated with TCs. They take place in the presence of strong winds and low atmospheric pressure. These surges usually result in extreme flooding and coastal inundations, causing large risks to human life as well as property. Tropical disturbances are classified according to their sustained winds. A tropical depression occurs when the wind speeds do not exceed 34 knots (63 km per hour). When the strength threshold moves beyond this, the system becomes a tropical storm and is assigned a designated name, e.g., Cyclone Amphan. When the sustained winds exceed 64 knots (118 km per hour), the storm is referred to as a hurricane; it earns classification as a hurricane in the Atlantic or East Pacific Oceans or a typhoon in the northern West Pacific. The destructive nature of tropical cyclones necessitates the development of automated detection and analysis systems to identify their characteristics and reduce the related risks.

In addition, much scientific interest has been directed towards exploring the trajectories of TCs and their relationships with other weather systems. Tropical storms greatly affect the air quality due to their strong winds and rain. They affect visibility greatly. The initial result of cyclones is a reduction in visibility that arises because of the increased suspension of dust, as well as fine particles, which may persist for some time. However, their ultimate results are usually favorable, creating clear skies and freshness in the atmosphere. These strong winds and intense showers help to remove air pollutants and atmospheric particles, leading to the enhanced dispersion of pollutants. In sum, tropical cyclones have temporary negative effects in affecting visibility but lead to long-term benefits.

The AQI is considered one of the main factors when evaluating air contamination, which can result in certain diseases and deterioration in the surrounding environment. The AQI captures complex air quality information as simple values, starting at zero and reaching up to five hundred, representing increasing air pollution. Furthermore, this numeric representation is divided into color-coded bands of good, moderate, unhealthy for sensitive groups, unhealthy, very unhealthy and hazardous, which quickly communicate health risks. AQI calculation involves the conversion of pollutant concentrations into sub-indices according to predetermined concentration breakpoints and choosing the major sub-index as the final AQI for a given location [4]. Therefore, the AQI acts as an immediate indicator of the potential health effects associated with air quality standards, contributing to public safety by informing citizens about the health risks of the existing environment. Beyond its utility as a public awareness tool, the AQI plays a pivotal role in developing policies and regulations. By monitoring AQI trends over time, authorities can pinpoint pollution sources, gauge the success of various remedial measures and develop targeted countermeasures to improve the air quality.

### 1.1. Limitations and Challenges

The urgent need to improve the air quality prediction and modeling accuracy during extreme weather events, especially cyclones, has been increasingly highlighted [5]. Limitations in air quality prediction during cyclones arise due to the complex and rapidly changing meteorological conditions, which can disrupt pollutant dispersion models and impact public health. Similarly, model limitations and performance issues highlight the need to refine the existing predictive models to account for the unique atmospheric dynamics of cyclonic systems, ensuring that they can capture air quality fluctuations effectively. Data challenges and generalization limitations point to the scarcity of comprehensive, high-resolution datasets that span various regions and cyclone intensities, which hinders models’ generalization and accuracy across different geographical contexts. Furthermore, the need for enhanced AQI prediction during cyclone events is critical, as more reliable forecasts can aid in timely decision-making for public safety, evacuation and resource allocation in vulnerable regions. Addressing these challenges will not only improve the accuracy of predictions but also strengthen our capacity to mitigate the health impacts of poor air quality during cyclonic events.

### 1.2. Contributions of This Work

In this work, the Literature Survey section addresses the challenges in predicting air quality during cyclones and discusses methods to improve the accuracy. It offers a thorough analysis of each method, providing valuable insights into the current state of air quality prediction during cyclones and the datasets used. This section also elucidates the specific limitations of existing works, which contributes to the proposal of the multimodal architecture.

To address the need for improved air quality prediction during cyclone events, the proposed work contributes by introducing a comprehensive architectural framework that combines multiple advanced models, including Convolutional LSTM (ConvLSTM), Real-ESRGAN, convolutional neural networks (CNNs) and an AQI regression model. Together, these components effectively tackle the challenges associated with predicting the wind speed and forecasting the air quality. ConvLSTM excels in capturing and learning spatiotemporal patterns, making it particularly suitable for the forecasting of weather-related phenomena such as the wind intensity. On the other hand, Real-ESRGAN significantly improves the quality of visual data, which enhances the accuracy of image sequence analysis, critical for environmental predictions [6]. The CNN model is essential in forecasting wind speeds, showcasing the effectiveness of deep learning methodologies in meteorology. Additionally, the AQI regression model offers valuable insights into air quality trends by predicting changes influenced by environmental conditions and meteorological events like tropical cyclones.

Moreover, this research highlights a crucial link between meteorological events, such as tropical cyclones, and natural ecosystems, providing a deeper understanding of their effects on the air quality and environmental conditions. The interdisciplinary nature of this research amplifies its significance for practical applications, supporting emergency response planning, environmental management and public health strategies related to air quality and meteorological risks. By merging meteorological data with cutting-edge deep learning techniques, this study enables more informed decision-making across various sectors, particularly in environmental health and disaster management, emphasizing the necessity of a comprehensive strategy to address these urgent challenges.

### 1.3. Organization of the Paper

In order to address issues with wind speed prediction and air quality forecasting, this work attempts to propose a comprehensive architecture that includes several advanced models, including Convolutional LSTM, Real-ESRGAN, CNNs, and AQI regression. Section 2 examines recent studies, emphasizing the techniques used, the precision attained and the drawbacks of the state-of-the-art methods. In Section 3, we outline the architecture’s initial configuration, including the datasets and mathematical foundations that our proposed system makes use of. The proposed multimodal technique is introduced in Section 4. Section 5 includes a detailed explanation of each phase in the research, beginning with the model setup and progressing to the validation methods. Section 6 summarizes the model’s findings and evaluates its performance. Finally, Section 7 concludes the paper by discussing future directions for research.

## 2. Background Work

In this section, we review recent works, highlighting the methodologies adopted, the accuracy achieved and their limitations. We organize them into two broad categories: (1) tropical cyclone detection and intensity prediction and (2) air quality prediction during cyclone events.

Furthermore, we present a comparative analysis as a crucial aid to our work. Each of the reviewed works is carefully examined and summarized in a tabulated format to showcase their contributions, as well as any limitations noted. Additionally, we acknowledge the datasets utilized in the previous research and present any overarching research gaps to conclude.

The detection of tropical cyclones (TCs) from satellite images has advanced significantly, with various models improving the accuracy and performance. Nair et al. achieved high precision (97.10%), specificity (97.59%) and accuracy (86.55%) with a deep learning pipeline that detected TCs in 67 out of 88 test images and avoided false positives in 81 out of 88 non-TC images. This pipeline was bolstered by a CNN classifier and a wind speed filter. However, the initial Mask R-CNN model encountered issues with false positives, which were later reduced by prioritizing accuracy over the F1 score. While the CNN classifier improved the overall outcomes, it also decreased the number of true positive detections, indicating that further refinement is necessary [7].

Building on similar concepts, Jena et al. developed a deep convolutional neural network (DCNN) model that effectively classified satellite cloud images as either cyclonic or non-cyclonic, achieving 94% accuracy. This model outperformed traditional machine learning methods by excelling in feature extraction for classification purposes. However, the large image sizes used in training resulted in prolonged processing times, and reducing the image size could impact the model accuracy. The authors suggested that advanced computing hardware and higher-resolution images could further improve the performance [8].

Similarly, Lam et al. explored the use of thermal infrared (TIR) instruments like IASI in a model that achieved average precision of 78.31% at an IoU threshold of 0.1. However, the precision dropped significantly to 31.05% at an IoU threshold of 0.5, reflecting challenges in achieving precise predictions. This study recommended improving the input data by exploring other IASI products and incorporating advanced data augmentation techniques to enhance the model performance [2].

Chang et al. introduced the TCICENet model, which used infrared satellite images and a cascading deep CNN for the classification and estimation of the cyclone intensity, achieving promising error metrics with an RMSE of 8.60 kt and MAE of 6.67 kt. The model was particularly effective with 170 × 170 pixel images but was limited to the Northwestern Pacific region and did not account for other crucial physical factors like wind shear and the ocean temperature, which are planned for future model integrations [9].

In the context of predicting typhoon movement, Wang et al. developed a CNN-based model that predicted the movement direction over the Northwestern Pacific, with a mean angle error of 27.8 degrees. They suggested that increasing the model’s complexity, such as adding convolutional and batch normalization layers, could reduce this error. However, the model’s small training dataset of 2250 images and its focus on the movement direction, and not the distance, restricted its broader application [10]. Wang et al. also introduced a two-step framework combining object detection and image processing techniques to locate TC centers in satellite infrared images. Nevertheless, the object detection model’s accuracy and circle selection in the Hough transform required further improvement [4].

Maskey et al. developed a CNN model that successfully transitioned into production for the estimation of a tropical cyclone’s intensity from satellite imagery. However, challenges remained, particularly due to the subjective nature of the Dvorak technique and the limited temporal frequency of passive microwave data compared to infrared. The difficulty in explaining the model’s decision-making process could also hinder its acceptance by experts [11].

In a different approach, Jin et al. proposed a three-step algorithm to locate tropical cyclone centers in synthetic aperture radar (SAR) images, particularly when TC eyes were not visible. While the inflow angle model outperformed the logarithmic spiral model, the algorithm’s reliance on accurate inflow angles and wind speeds posed limitations, and the spiral model’s inability to fit all rain band shapes further reduced its broader applicability [3].

Huang et al. developed the MMSTN model, which captured trajectory–intensity relationships to predict tropical cyclone paths. While outperforming other methods in short-term predictions, the model struggled in learning cyclones’ mutability from historical data alone, indicating the need for the integration of additional physical constraints. Zhou et al. developed the GC-LSTM model, combining graph convolutional and long short-term memory networks to classify and predict typhoon levels from satellite cloud images. Despite showing promise, the model’s evaluation was limited to a single image processing algorithm (GCN), and the study did not test it on large-scale real-world datasets [12].

When analyzing the air quality during cyclone events, Chen et al. focused on the Guangdong–Hong Kong–Macao Greater Bay Area (GBA), raising concerns about the model’s ability to generalize to other regions with different climates. Their study also highlighted the need to incorporate local emission sources like industrial activities and vehicle emissions into future models to improve the accuracy [13]. Similarly, Liu et al. emphasized the importance of employing more advanced optimization techniques, such as genetic algorithms, to better address air quality index (AQI) fluctuations and improve models’ flexibility through adaptive approaches [14].

Dehshiri et al. faced challenges in their model due to the limited temporal resolution of the satellite data, which impacted the real-time air quality assessments. Additionally, a negative bias in the Sentinel-5P NO2 data affected the reliability of the predictions, particularly in areas with uneven air quality monitoring station distributions. They suggested expanding the monitoring network to address these gaps [15]. Dong et al. further underscored the importance of higher-resolution data to improve air quality predictions, especially in understanding how extratropical cyclones (ETCs) interact with air pollution. Their study called for a more detailed exploration of these interactions to refine the predictive models during ETC events [16].

### Comparative Analysis

Numerous studies have examined cyclone tracking and air quality prediction methods, but many still encounter limitations, emphasizing the need for advancements. In particular, the integration of air quality data during cyclones remains underdeveloped. The works in Table 1 and Table 2 highlight key issues involving model accuracy, data limitations and dataset constraints, all critical in improving cyclone and air quality forecasting systems [17].

Model Limitations and Performance Issues: Nair et al. [7] employed CNN and Mask R-CNN models, but early versions yielded many false positives, revealing the need for better model refinement. Jena et al. [8] applied a deep convolutional neural network (DCNN) for cyclone prediction but experienced lengthy training periods due to large image sizes, negatively impacting its accuracy. Balancing the image resolution with processing efficiency remains a challenge. Similarly, Wang et al. [10] and Lam et al. [2] also used CNN-based approaches for cyclone path prediction. However, these models struggled with small training datasets and declining performance at higher IoU thresholds, limiting their real-world applicability. These examples illustrate that while deep learning models offer promise, they continue to face challenges in accuracy, processing speed and dataset generalization.

Limitations in Air Quality Prediction During Cyclones: Predicting the air quality during cyclone events has its own complexities. Chen et al. [13] and Liu et al. [14] noted that their air quality prediction models struggled with regional limitations, such as being confined to the Guangdong–Hong Kong–Macao Greater Bay Area (GBA), and required real-time meteorological data for more accurate results. These studies suggest that many models are either too region-specific or lack the integration of real-time air quality data, thus limiting their effectiveness in fast-changing cyclone events.

Data Challenges and Generalization Limitations: Data availability and quality are recurring problems in the reviewed works. Chang et al. [9] and Maskey et al. [11] leveraged satellite imagery, but these models relied heavily on specific data types, like infrared satellite images, or struggled to fully explain the rationale behind the cyclone intensity classifications [18]. Furthermore, air quality datasets, such as those used by Anggraini et al. [15], are limited by negative biases in NO_2_ data and insufficient ground monitoring stations, resulting in inaccurate air quality predictions. Dong et al. [16] also emphasized the need for higher-resolution data to enhance the understanding of extratropical cyclone and pollution interactions—an area that remains under-researched.

Need for Enhanced AQI Prediction During Cyclone Events: The limitations identified in previous studies demonstrate a critical need for models that integrate air quality index (AQI) predictions into cyclone forecasting [19]. Cyclones influence the air quality by dispersing pollutants or worsening the pollution levels through altered wind patterns, but the current models often overlook these interactions. Most studies suffer from a lack of real-time, high-resolution data, which, along with the complexity of air quality forecasting in cyclone-prone areas, highlights a significant research gap.

In order to improve AQI prediction, the model should include a wide range of real-time data, such as satellite, meteorological and pollution data. It should also be able to adapt to the changing conditions caused by cyclones. Addressing these issues is crucial in providing more precise AQI forecasts, which can enhance public health and emergency responses during severe weather events.

Table 2 summarizes the key datasets, including satellite images, air quality data and meteorological information, used in various studies focused on tropical cyclones and air quality assessments. Lam et al. [2] used IASI data with 1985 images covering 50 channels, while Wang et al. [4] utilized Himawari-8 satellite data with 2250 images focusing on 97 typhoons. Nair et al. [7] analyzed data from the Meteosat satellite in six-hour intervals from 2001 to 2016, and Maskey et al. [11] employed GOES satellite data at 15 min intervals over two decades. Chang et al. [9] used multiple satellite sources, augmenting 19,451 images from 1001 tropical cyclones. Zhou et al. [1] relied on Fengyun satellite data and best-track data, while Jena et al. [8] used 8436 meteorological records for cyclone classification. Similarly, Huang et al. [12] used Himawari-8 data from 2010–2019, classifying images into various typhoon categories. On the air quality front, Yilin Chen et al. [13] combined typhoon and air quality data from 39 stations, while Chunhao Liu et al. [14] provided hourly air quality measurements. Tania Septi Anggraini et al. [20] used global air quality data from 425 stations alongside satellite data, and Peiyun Dong et al. [16] integrated meteorological data from China’s monitoring center with satellite models. The proposed method utilizes HURSAT data (1978–2009) and meteorological records from Tutiempo, offering a comprehensive view of tropical cyclones’ characteristics across several decades.

**Table 1:** Comparative analysis of cyclone and air quality prediction models.

Work by	Model Type	Performance	Key Strengths	Limitations	Data Sources	Applications
Nair et al. [7]	CNN Classifier with Wind Speed Filter	Precision: 97.10%, Specificity: 97.59%, Accuracy: 86.55%	High precision, low false positives	Reduction in true positives due to prioritizing accuracy over F1 score	Satellite images and wind speed (visible and infrared)	TC detection in 67 out of 88 test images
Jena et al. [8]	Deep CNN (DCNN)	94% accuracy	Effective feature extraction, outperformed traditional ML	Prolonged training time with large images, potential accuracy drop with smaller image sizes	Satellite cloud images	Cyclonic vs. non-cyclonic image classification
Lam et al. [2]	CNN + IoU for Object Detection	Precision: 78.31% (IoU 0.1), 31.05% (IoU 0.5)	Demonstrated utility of TIR instruments (IASI)	Poor performance at higher IoU thresholds, need for better input data	Thermal infrared (TIR) instruments like IASI	Object detection in thermal images, cyclone detection
Chang et al. [9]	TCICENet (Cascading Deep CNN)	RMSE: 8.60 kt, MAE: 6.67 kt	Optimal performance with 170 × 170 pixel images	Limited to Northwest Pacific, excluded key physical factors like wind shear	Infrared satellite images	Cyclone intensity classification and estimation
Wang et al. [10]	CNN-Based Model for Movement prediction	Mean angle error: 27.8 degrees	Focused on movement direction prediction	Small training dataset (2250 images), did not predict movement distance	Satellite infrared images	Typhoon movement direction prediction
Wang et al. [4]	Two-Step Framework (Object Detection + Image Processing)	TC center detection using object detection and Hough transform	Refines TC center location using morphological characteristics	Inaccuracy in object detection model, circle selection using Hough transform	Satellite infrared images	TC center detection from satellite images
Maskey et al. [11]	CNN-Based Intensity Estimation	Successfully transitioned into production	Good transition into real-world usage	Challenges with subjective Dvorak technique, limited microwave data, model explainability	Satellite imagery (infrared and microwave)	Cyclone intensity estimation
Jin et al. [3]	Three-Step Algorithm (SAR Image Analysis)	Inflow angle model outperformed logarithmic spiral model	Effective at locating TC centers without visible eyes	Reliance on actual inflow angles and wind speed, limited fit for all rain band shapes	Synthetic aperture radar (SAR) images	TC center location in absence of visible cyclone eyes
Zhou et al. [1]	GC-LSTM (Graph Convolutional + LSTM Network)	Typhoon level prediction	Hybrid architecture combining GCN and LSTM	Focus on single image processing algorithm, lacked real-world dataset validation	Satellite cloud images	Typhoon classification and prediction
Huang et al. [12]	MMSTN Model (Trajectory intensity prediction)	N/A	Accurate short-term TC trajectory and intensity prediction	Struggles with learning from historical data, lacks external constraints	Historical cyclone data	Short-term prediction of cyclone paths
Chen et al. [13]	Air Quality Prediction Model	Focused on GBA region, localized air quality predictions	Highlighted local emission sources like industrial activities	Limited generalizability outside the GBA region	Satellite imagery, local emission sources	Air quality prediction during cyclone events
Liu et al. [14]	Genetic Algorithm Optimization for AQI Fluctuations	Suggested advanced optimization techniques for better performance	Proposed genetic algorithms and adaptive approaches for model flexibility	Need for more flexible hidden layer nodes to adapt to different conditions	Air quality data, satellite imagery	Addressing AQI fluctuations during cyclone events
Anggraini et al. [15]	Air Quality Prediction using Sentinel-5P Data	Model struggled with Sentinel-5P NO_2_ biases, affecting predictions	Highlighted real-time AQI assessment limitations	Temporal resolution of satellite data, uneven air quality monitoring station distribution	Sentinel-5P NO_2_ satellite data	Real-time air quality assessment, AQI prediction
Dong et al. [16]	ETC–Air Quality Interaction Prediction	Stressed need for higher-resolution data to improve predictions	Explored interactions between extratropical cyclones and air pollution	Data quality and availability constraints, focused on extratropical cyclone events	Satellite data, environmental data	Air quality prediction during extratropical cyclone (ETC) events

**Table 2:** Summary of datasets used in tropical cyclone and air quality studies.

Work by	Dataset	No. of Images/ Instances	Class Labels	Balanced	Remarks
Lam et al. [2]	IASI Data	1985	Tropical cyclone stages	N/A	Bounding boxes from HURDAT2; dataset covers 50 channels for better feature extraction
Wang et al. [10]	Himawari-8 Satellite	2250	Typhoon levels (Channels 13 and 15 of AHI)	Likely imbalanced (97 typhoons from 2015–2018)	Only 97 typhoons in the dataset over three years; potentially imbalanced due to limited events
Nair et al. [7]	Meteosat (MVIRI)	-	Cyclone vs. Non-Cyclone	N/A	Six-hour intervals; long-term dataset covering 2001–2016; COCO format used
Maskey et al. [11]	GOES-8 to GOES-16	-	Cyclone intensity classes	Likely imbalanced	Dataset includes 15 min intervals from 2000 to 2019; HURDAT2 reanalysis used
Xu et al. [6]	Multiple Satellites	19,451	Tropical cyclone intensity	Likely balanced	1001 TCs with data augmentation through rotation and noise; dataset is designed to handle imbalances
Zhou et al. [1]	Fengyun Satellites	-	Typhoon classification (Best-track data)	Likely balanced	Uses best-track data from CMA, aiming to balance typhoon levels
Jena et al. [8]	Meteorological	8436	Cyclone vs. Non-Cyclone	Balanced	Dataset split into train, test and validation sets for balanced training
Huang et al. [12]	Himawari-8	4100	Tropical depression, typhoon, strong typhoon, super typhoon	Not mentioned	Classified from 2010 to 2019; covers different intensity levels
Jin et al. [3]	SAR Images	6	Cyclone intensity (different sensors)	Not balanced	Very small dataset, limiting the model’s ability to generalize
Huang et al. [12]	CMA Best Track	-	Cyclone characteristics	Likely balanced	Six-hourly data on latitude, longitude, pressure, wind speed
Yilin Chen et al. [13]	Air Quality, Typhoon and Meteorological Data	-	Air quality metrics and cyclone events	Balanced	Dataset from 39 monitoring stations; integrates air quality and meteorological data
Chunhao Liu et al. [14]	Air Quality Dataset	-	Hourly pollutant data	Likely balanced	Comprehensive pollutant data available on GitHub
Tania Septi Anggraini et al. [20]	Satellite and Ground-Based Data	-	Air quality metrics	Likely imbalanced	Global dataset from 2019 to 2021, AQI data from 425 stations globally
Peiyun Dong et al. [16]	Air Quality and Meteorological Data	-	Air quality and cyclone interactions	Likely imbalanced	Data from China National Environmental Monitoring Center; HYSPLIT model and CALIPSO satellite data
Method Proposed	HURSAT Dataset	Global satellite imagery of TC	Tropical cyclone characteristics	Likely balanced	Global satellite data on tropical cyclones from 1978 to 2009; Gridded NetCDF format
	Tutiempo Meteorological Data	Contains hourly or daily observations	Raw meteorological variables; no class labels associated	Likely balanced	The dataset is sourced from global meteorological stations and is useful in tracking historical weather patterns and events.

## 3. Setting up the Multimodal Model

In this section, we describe the initial process of setting up the architecture, i.e., the datasets used and the mathematical prerequisites in each of the processes.

### 3.1. Datasets Fueling the Model

In our work, we use the following datasets for the respective purposes.

#### 3.1.1. Satellite Dataset

The satellite data were obtained from the Hurricane Satellite (HURSAT) dataset run by means of the National Centers for Environmental Information. This has been utilized because it is the primary source for satellite imagery. It is a collection of satellite statistics on tropical cyclones globally, supplying a complete and reliable source of records for researchers and organizations studying such storms. It accommodates gridded statistics saved in a NetCDF layout, encompassing a wide variety of parameters including cloud insurance, the sea floor temperature and the wind velocity, derived from observations made through various geostationary satellites [8,20].

The HURSAT task first centered on HURSAT-B1, which encompasses raw satellite observations spanning from 1978 to 2009. However, it has been increased to include information from other satellite sources, together with HURSAT-AVHRR and HURSAT-MW, providing records with varying temporal and spatial resolutions. The HURSAT dataset has been validated for a multitude of research endeavors, allowing scientists to delve into the complex dynamics of tropical cyclones and their long-term influences. These meteorological satellites provide a valuable supply of information to track the movement, length and severity of these storms throughout their durations, illuminating the factors involved. This knowledge is vital for accurate forecasting that can predict the tracks and intensities of tropical cyclones, supplying essential early warnings to coastal groups that are likely to be impacted by these storms [9,11,17].

Moreover, the HURSAT dataset plays a pivotal role in formulating powerful mitigation techniques, guiding the identification of areas liable to tropical cyclone damage and facilitating the development of evacuation plans and property safety measures. In essence, the HURSAT dataset stands as a cornerstone in comprehending and responding to tropical cyclones. The information supplied for the storms is complete and dependable, because it enables researchers and businesses to delve into each of the storms themselves and to forecast the paths of these storms, verify any viable impacts and implement vital measures to alleviate any negative outcomes. The HURSAT dataset has undoubtedly revolutionized our knowledge of tropical cyclones, fostering more proactive techniques to mitigate their impacts and safeguard coastal groups worldwide.

#### 3.1.2. Meteorological Dataset

This study utilized meteorological data sourced from Tutiempo, a comprehensive weather website, providing a valuable platform for climate record evaluation. Its sizable real-time and historical information encompasses a wide range of meteorological parameters and global regions, provides a vast supply of data for researchers and individuals alike. The consumer-friendly interface and intuitive visualizations facilitate records’ exploration and pattern determination, enabling researchers to delve into complex weather dynamics and climatological developments. Additionally, Tutiempo’s seamless cell compatibility guarantees accessibility and user friendliness, allowing for real-time climate monitoring and evaluation. Whether investigating long-term climate patterns, assessing the accuracy of climate forecasts or correlating climate data with different environmental elements, Tutiempo emerges as a flexible and effective tool for climate information analysis.

### 3.2. Mathematical Preliminaries

Evaluating the accuracy of predictions ensures the effectiveness, reliability and optimization of models, making this an essential aspect of data-driven decision-making. To validate and evaluate the respective multimodal models, the following mathematical constructs are used in our work.

#### 3.2.1. Absolute Error

The absolute error is a metric for the evaluation of the accuracy of predictions and is calculated as [21]

(1) $$AE_{i}=\left|y_{i}-{\hat{y}}_{i}\right|$$

where

- $AE_{i}$ is the absolute error for the *i*th prediction;
- $y_{i}$ are the actual values;
- ${\hat{y}}_{i}$ are the predicted values.

#### 3.2.2. Mean Absolute Error (MAE)

A widely used metric for the evaluation of model performance is the mean absolute error (MAE), which is computed by averaging the absolute errors across all predictions. It can be represented as [21,22]

(2) $$\mathrm{Mean}\;\mathrm{Absolute}\;\mathrm{Error}\;\left(\mathrm{MAE}\right)=\frac{1}{n}\sum_{i=1}^{n}AE_{i}$$

where

- *n* is the total number of predictions.

#### 3.2.3. Analyzing the Category

The relationship between the sample size and model prediction accuracy can be observed through the distribution of the absolute errors across different categories. For a specific category $C_{j}$ with $n_{j}$ samples, the category-specific $MAE_{j}$ can be analyzed as follows:

(3) $$MAE_{j}=\frac{1}{n_{j}}\sum_{i=1}^{n_{j}}\left|y_{ij}-{\hat{y}}_{ij}\right|$$

where

- $MAE_{j}$ is the mean absolute error for category $C_{j}$;
- $y_{ij}$ are the actual values for category $C_{j}$;
- ${\hat{y}}_{ij}$ are the predicted values for category $C_{j}$;
- $n_{j}$ is the number of samples in category $C_{j}$.

Lower $n_{j}$ values generally lead to higher $MAE_{j}$, indicating that categories with fewer samples may result in larger prediction errors. In contrast, categories with larger sample sizes tend to exhibit lower absolute errors.

#### 3.2.4. Root Mean Squared Error (RMSE)

The root mean squared error (RMSE) provides another measure of the differences between the predicted and actual values [23]:

(4) $$RMSE=\sqrt{\frac{1}{n}\sum_{i=1}^{n}{\left(AE_{i}\right)}^{2}}$$

#### 3.2.5. Multimodal Evaluation Techniques

In the context of evaluating multimodal models [24], it is essential to compare and assess the performance of multiple models to ensure that the most accurate and reliable predictions are being made. The following evaluation tools are commonly employed.

##### Coefficient of Determination ($R^{2}$)

The performance of the model is evaluated using the $R^{2}$ score, which measures how well the predicted values match the actual values. The $R^{2}$ score is defined as [25]

(5) $$R^{2}=1-\frac{\sum_{i=1}^{n}{\left(y_{i}-{\hat{y}}_{i}\right)}^{2}}{\sum_{i=1}^{n}{\left(y_{i}-\bar{y}\right)}^{2}}\;$$

where

- $y_{i}$ are the actual values;
- ${\hat{y}}_{i}$ are the predicted values;
- $\bar{y}$ is the mean of the actual values;
- *n* is the number of data points.

##### Cross-Validation Score

To assess the model’s robustness, cross-validation is performed using 5 folds (k=5). The cross-validation score is calculated as the average $R^{2}$ score over *k* folds [26]:

(6) $$\text{CV\_Score}=\frac{1}{k}\sum_{i=1}^{k}R_{i}^{2}$$

where

- *k* is the number of folds (here, k=5);
- $R_{i}^{2}$ is the $R^{2}$ score for fold *i*.

## 4. Proposed Multimodal Methodology

The comparative analysis, based on the observations from Table 1, highlights the necessity for a tailored model within the proposed multimodal approaches. By employing a multimodal framework, we integrate ConvLSTM, GANs and CNNs to provide predictive insights into different dimensions of tropical cyclones’ behavior. Additionally, we use a machine learning regression technique to forecast the air quality index (AQI), combining multiple methods to improve the accuracy across these environmental phenomena.

**Figure 1:**
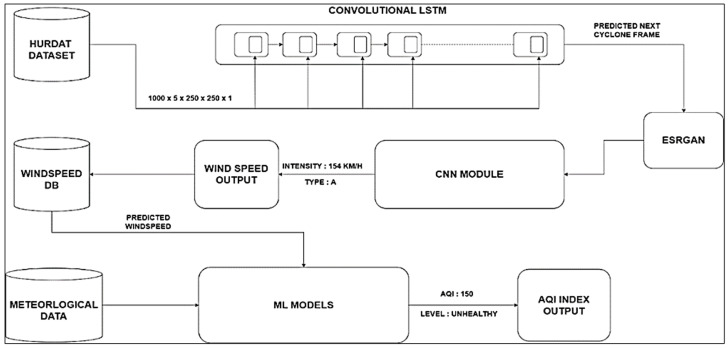
Architecture of the proposed model.

Furthermore, this paper presents an AQI analysis using regression modeling with predictive outcomes regarding how the air quality is expected to change at different stages of a tropical storm. The first stage involves ConvLSTM, analyzing images from ground-based and satellite monitoring stations. With this technique, it becomes possible to extract the spatial–temporal patterns of cyclogenesis. The use of a pre-trained wind speed prediction CNN model to aid cyclone categorization follows. An advanced tool interprets the CNN model’s predictions as they relate to the type of damage that the cyclone might cause. In parallel, GANs are integrated, specifically Real-ESRGAN, for image enhancement, specializing in refining the illustration of cyclonic events. This multimodal framework allows for a holistic evaluation of tropical cyclones, combining the strengths of various neural network architectures. This study uses various datasets, including meteorological data and satellite images, alongside machine learning methodologies, which enables the study of short-term and long-term changes with respect to the air quality and cyclones. This serves to improve the predictive models for tropical cyclones and enables a greater understanding of the effects of cyclones on the environmental conditions. The proposed architecture is illustrated in Figure 1. In the next section, we present each process with its detailed algorithm and discuss the validation of the model in detail.

## 5. Process Breakdown: Model Configuration and Performance Assessment

This section offers a thorough explanation of each process involved in the research. It begins by outlining the steps involved in setting up the model, followed by a detailed description of the validation procedures. Here, the system used is equipped with an Intel i7 processor, which offers robust performance to handle complex computations and multitasking. It also features an NVIDIA graphics card, enabling the efficient processing of graphically demanding tasks. Additionally, the system includes 16 GB of RAM, ensuring seamless operation during intensive data analysis and concurrent software execution.

Finally, this paper delves into the methods used to evaluate the processes, ensuring a complete understanding of each phase, which is presented in detail in the next section.

### 5.1. ConvLSTM

An integrated approach leveraging convolutional long short-term memory (ConvLSTM) networks, image processing techniques and model evaluation methodologies was undertaken.

#### 5.1.1. Setting up the Model

The foundation of the work relied on a diverse set of libraries and dependencies.

**Figure 2:**
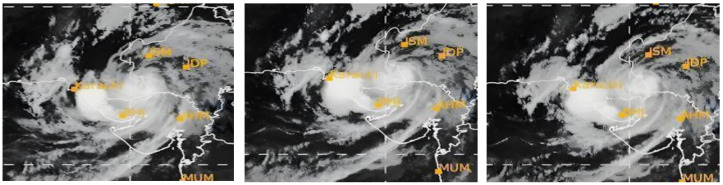
Image Please cite the figure before it. sequence of TCs.

To test the model, the input consists of image sequences of TCs depicted in Figure 2.

**Figure 3:**
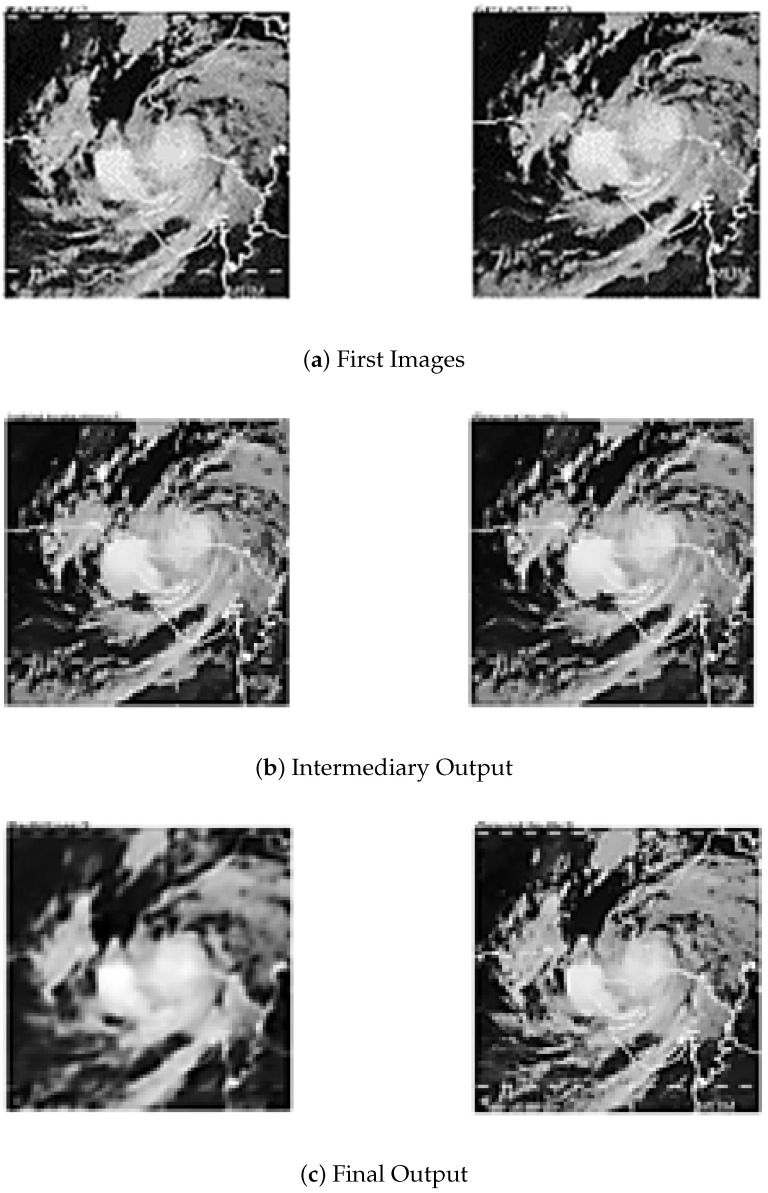
Predicted images generated by ConvLSTM vs. ground truth.

Figure 3 provides a comparison between the actual ground truth and the predicted TC images generated by the model, as illustrated by Algorithm 1.
**Algorithm 1** Preprocessing and ConvLSTM Model Development**Libraries**: Import required libraries—TensorFlow, Keras, NumPy, OpenCV.**Input**: Image sequences (Ex. in Figure 2)**Output**: Serialized ConvLSTM model and predictions  1:**Step 1: Load Images**  2:Load image sequences $\left\{I_{1},I_{2},\cdots ,I_{n}\right\}$ from the input folder  3:**Step 2: Preprocessing**  4:Convert images to grayscale:  5:Resize images to 50×50 pixels and reshape them for ConvLSTM input.  6:Normalize pixel values:
$I_{\mathrm{norm}}=\frac{I_{\mathrm{gray}}}{255.0}$  7:**Step 3: Model Construction**  8:Construct ConvLSTM model with convolutional layers, batch normalization and a Conv3D layer with sigmoid activation:
$$\hat{y}=\sigma \left(W\cdot X+b\right)$$
where $\hat{y}$ is the prediction, *W* are the weights, *X* is the input and *b* is the bias term.  9:**Step 4: Training**10:Define the loss function as the Mean Squared Error (MSE), as mentioned in Equation (2)11:Train the model12:**Step 5: Save Model**13:Serialize the trained model.14:**Step 6: Evaluation**15:Compare predicted images $\hat{y}$ with ground truth images *y*.

#### 5.1.2. Validation and Evaluation of the Model

Subsequently, a model evaluation protocol was performed, including a performance appraisal on the test data and plots of the training and validation loss, MAE and RMSE. Furthermore, this study delved into image generation and animation techniques, utilizing the trained ConvLSTM model to produce a sequence of images, which were subsequently combined into an animated GIF for a thorough visual evaluation.

### 5.2. CNN

In the convolutional neural network (CNN) prediction stage of our research, an important element is the seamless integration of a pre-trained CNN model, meticulously trained on an extensive dataset to identify and capture the preferred functions and patterns inherent in tropical cyclone images. The process is illustrated in Algorithm 2.
**Algorithm 2** Wind Speed Prediction and Tropical Cyclone Classification using Pre-Trained CNN**Libraries Required**: TensorFlow, OpenCV, Python Imaging Library (PIL), NumPy**Input**: Image *I***Output**: Predicted wind speed and tropical cyclone category  1:**Step 1: Load Pre-Trained Model**  2:**Step 2: Image Retrieval and Preprocessing**  3:Retrieve the image *I*  4:Convert the image to grayscale  5:Resize the image to 250×250 pixels  6:Reshape the image to (1,250,250,1) for model input:
$I_{\mathrm{reshaped}}\leftarrow \mathrm{reshape}\left(I_{\mathrm{resized}},\left(1,250,250,1\right)\right)$  7:Normalize the pixel values by dividing by 255:
$I_{\mathrm{norm}}=\frac{I_{\mathrm{reshaped}}}{255.0}$  8:**Step 3: Wind Speed Prediction Using CNN Model**  9:Feed the normalized image into the CNN model:
$$\hat{y}\leftarrow \mathrm{model}.\mathrm{predict}\left(I_{\mathrm{norm}}\right)$$
where $\hat{y}$ is the predicted wind speed.10:**Step 4: Wind Speed Scaling**11:Scale the predicted wind speed $\hat{y}$ by a factor of 10:
${\hat{y}}_{\mathrm{scaled}}=10\times \hat{y}$12:**Step 5: Cyclone Category Classification**13:Categorize the wind speed based on the Saffir–Simpson scale:
$$\mathrm{Category}\;1:\;74\leq {\hat{y}}_{\mathrm{scaled}}<95\;\mathrm{mph}$$
$$\mathrm{Category}\;2:\;96\leq {\hat{y}}_{\mathrm{scaled}}<110\;\mathrm{mph}$$
$$\mathrm{Category}\;3:\;111\leq {\hat{y}}_{\mathrm{scaled}}<129\;\mathrm{mph}$$
$$\mathrm{Category}\;4:\;130\leq {\hat{y}}_{\mathrm{scaled}}<156\;\mathrm{mph}$$
$\mathrm{Category}\;5:\;{\hat{y}}_{\mathrm{scaled}}\geq 157\;\mathrm{mph}$14:**Step 6: Visualize Results**15:Display the original resized image along with the predicted wind speed and cyclone category

#### 5.2.1. Setting up the Model

**Figure 4:**
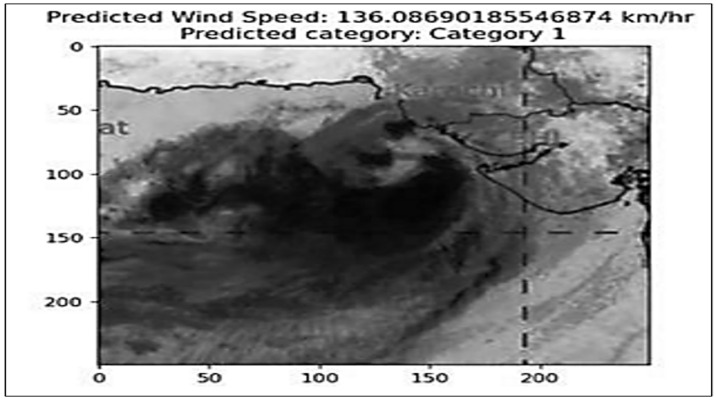
Wind speed and category predicted by CNN.

The incorporation of this pre-trained model and its workings are illustrated in Algorithm 2. Thus, the original resized image is visually represented along with key facts derived from the CNN model. This includes the predicted wind speed, a numerical illustration of its meteorological importance and the ascertained class of the tropical cyclone, as shown in Figure 4. The visual representation serves as an intuitive and on-hand method of conveying the model’s predictions, emphasizing the CNN’s efficacy in predicting the meteorological parameters and categorizing tropical cyclones based on image data. This system underscores the model’s robustness and its capacity in real-world meteorological situations.

The Saffir–Simpson scale for comparisons increases the validity of the CNN model’s predictions. The Saffir–Simpson scale is a hurricane wind scale that estimates the potential property damage caused by hurricanes. It is based on the hurricane’s sustained wind speed and categorizes hurricanes into five categories, Category 1, Category 2, Category 3, Category 4 and Category 5, as mentioned in Algorithm 2.

#### 5.2.2. Validation and Evaluation of the Model

The model can predict the wind speed category, which correlates with the known meteorological scale, making the model effective. This even provides some qualitative predictions that align well with the already standardized approach commonly used by scientists. The above aspect strengthens the model’s predictions and confirms its relevance in classifying wind speeds.

**Figure 5:**
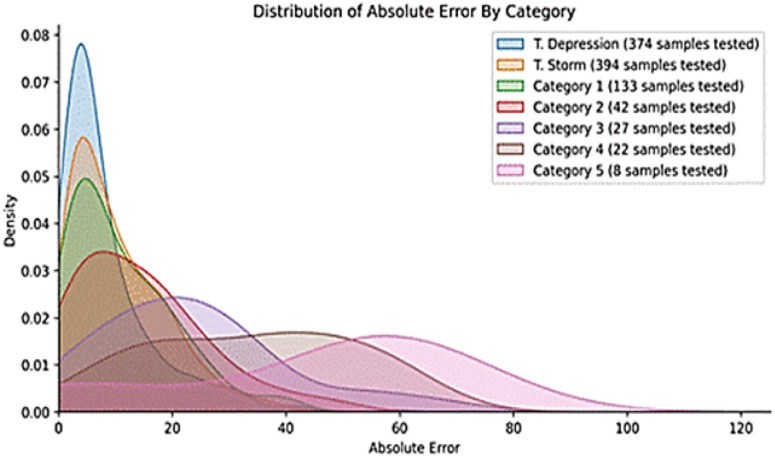
Distribution of absolute error by category.

Figure 5 portrays the distribution of the absolute error across the TC categories for the CNN model, ranging from a tropical depression to a category 5 cyclone.

These categories are first divided on the basis of the wind speeds. The absolute error serves as a metric for the evaluation of the model’s ability to predict the actual data values accurately. Lower absolute error values translate to better model fitting. The graph reveals a general trend of lower absolute errors for categories with more samples. This stems from the model’s enhanced capacity to learn about these categories from the available data. For instance, the category with the most samples (T. Depression) exhibits the lowest absolute error. Conversely, categories with fewer samples tend to exhibit higher absolute errors. This arises from the limitations imposed by fewer training data. For example, the category with the fewest samples (category 5) demonstrates the highest absolute error. Overall, the graph suggests that the model effectively fits the data, even for categories with limited samples. However, the absolute error generally remains lower for categories with more data.

### 5.3. GAN

A detailed demonstration of Real-ESRGAN, an advanced image restoration model installed on the Google Colab platform, is presented.

#### 5.3.1. Setting up the Model

The Real-ESRGAN model is constructed in our work as mentioned in Algorithm 3. Snapshots of the Real-ESRGAN output are observed by extracting crucial image properties, such as the width and height. Additionally, these output images are systematically stored for further evaluation.
**Algorithm 3** Real-ESRGAN Inference Pipeline**Libraries Required**: Git, Python, Real-ESRGAN, OpenCV**Input**: Low-resolution image $I_{\mathrm{low}}$**Output**: High-resolution image $I_{\mathrm{high}}$  1:**Step 1: Setup**  2:Clone the repository and install dependencies:
gitcloneReal−ESRGAN,pipinstall−rrequirements.txt  3:**Step 2: Image Upload**  4:Prepare upload folder:
rm-rfupload/,mkdirupload/  5:**Step 3: Inference**  6:Run the inference script:
$$I_{\mathrm{high}}=\mathrm{SR}\left(I_{\mathrm{low}},f=4\right)\;\left(\mathrm{face}\_\mathrm{enhance}\;\mathrm{optional}\right)$$
where *f* is the upscaling factor.  7:**Step 4: Visualization**  8:Compare input and output images:
$$\mathrm{display}(I_{\mathrm{low}},I_{\mathrm{high}})$$Print properties of $I_{\mathrm{high}}$  9:**Step 5: Download Results**10:Compress and download results:
zip Real−ESRGAN_result.zip

#### 5.3.2. Validation and Evaluation of the Model

The subsequent section restates the visualization codes, including functions for image display, input–output pairs representation in reading and visualization. Consistent with the previous section, the script prints image properties for a greater understanding of the effects that Real-ESRGAN exhibits towards the input and output images. In essence, our research offers an extensive and illuminating examination of Real-ESRGAN, covering all aspects from setting up image upload to inference, visualization and image download, resulting in a wider discussion on image restorative methodologies and practice. The flow of the work in this model is illustrated in Algorithm 3.

### 5.4. Regression Models

This system revolves around machine learning tasks for AQI prediction. Machine learning models, specifically the random forest regressor and XGBoost regressor, are utilized. A comprehensive Python script has been presented for the analysis of air quality data and prediction of PM2.5 concentrations [27]. The overall process is illustrated in Algorithm 4.
**Algorithm 4** ML Pipeline for AQI Prediction and Feature Importance**Libraries Required**: Pandas, Seaborn, Matplotlib, Scikit-learn**Input**: Meteorological data from CSV files**Output**: AQI predictions and feature importance  1:**Step 1: Data Loading**  2:Load CSV files into DataFrame $df_{\mathrm{final}}$:
$$df_{\mathrm{final}}=\text{pd.concat}([df_{1},df_{2},\cdots ,df_{n}])$$
  3:**Step 2: Data Exploration**  4:Visualize relationships with pair plot and correlation heatmap:  5:**Step 3: Model Training**  6:Define features *X* (e.g., visibility, temperature) and target *y* (AQI):
X={features},y=AQI  7:Train models like linear regression and extra trees regressor:
$\hat{y}=X\beta \;(\mathrm{for}\;\mathrm{linear}\;\mathrm{regression})$  8:Evaluate models with metrics such as MSE, as mentioned in Equation (1)  9:**Step 4: Feature Importance**10:Calculate and visualize feature importance:
$I_{j}=\frac{1}{n_{\mathrm{trees}}}\sum_{i=1}^{n_{\mathrm{trees}}}I_{j}^{i}$11:Plot top 10 important features

#### 5.4.1. Setting up the Model

**Figure 6:**
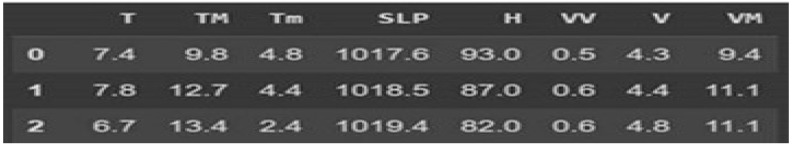
Data frame of the meteorological dataset.

**Figure 7:**
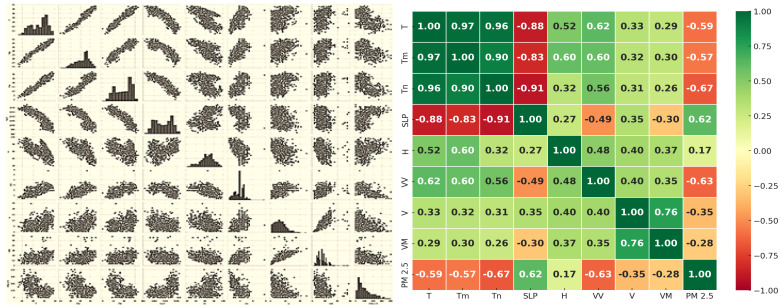
Pair plot and correlation plot between the meteorological variables.

The regression model under the multimodal process is constructed as illustrated in Algorithm 4. The preprocessing starts with combining the data from multiple CSV files into a unified data frame, as displayed in Figure 6. The meteorological variables explored are shown in Table 3. Then, it thoroughly explores the dataset using visualization, including a pair plot to show the relationships between the variables and a correlation heatmap plot to show how the features are related to each other or the target variable, as illustrated in Figure 7. The features of visibility, temperature and pressure exhibit the strongest correlations with the AQI. Furthermore, the visibility is internally dependent on the wind velocity.

**Figure 8:**
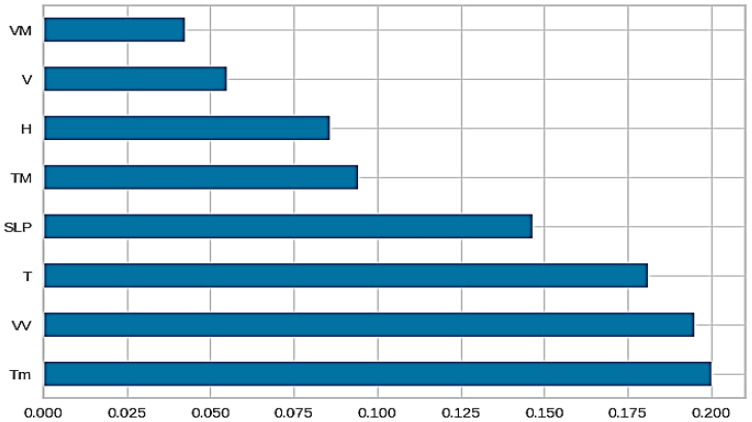
Feature importance of factors affecting air quality.

The script then employs machine learning techniques, including linear regression, extra trees regression and random forest regression, to explore the relationships between TCs and the AQI. It also visualizes the feature importance to gain insights into the factors influencing the air quality in the area of impact, as presented in Figure 8. Feature importance is a technique in machine learning that assigns a score to each feature in a dataset based on its relevance or contribution to the prediction of the output variable. In this context, the higher the score assigned to a feature, the more significant its impact on the prediction of the target variable. The calculation of the feature importance is often inherent in tree-based regressors. For this particular analysis, the extra trees regressor is employed to extract the top 10 features from the dataset.

#### 5.4.2. Validation and Evaluation of the Model

The dataset is split into training and testing sets, with 70% of the data used for training and 30% for testing. Then, an instance of the random forest regressor is created, and the model is trained on the training data (X_train, y_train). The coefficient of determination ($R^{2}$) is determined to evaluate the model’s performance on the training and test sets. $R^{2}$ is a measure of how well the predicted values match the actual values. It ranges from 0 to 1, where 1 indicates a perfect fit. The $R^{2}$ value observed for the training dataset is 0.97 and that for the test dataset is 0.81. Cross-validation is performed using five folds (cv = 5) to assess the model’s performance in a more robust manner. The mean of the cross-validation score achieved is 0.72. Hyperparameter tuning is performed for the random forest model using a randomized search CV.

**Figure 9:**
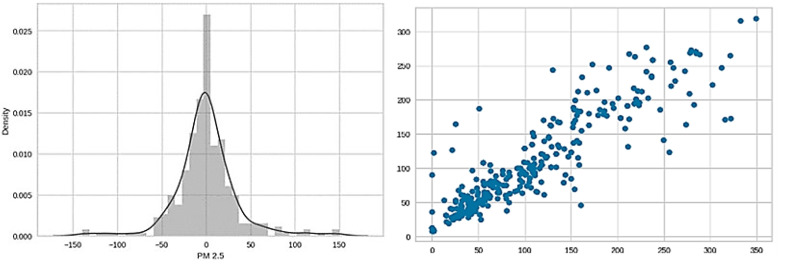
Distribution plot and scatter plot visualizing the differences between the actual and predicted values.

The model with the best hyperparameters is used to obtain predictions on the test set. A distribution plot and a scatter plot are created to visualize the differences between the actual and predicted values, as illustrated in Figure 9. The mean absolute error (MAE), mean squared error (MSE) and root mean squared error (RMSE) are calculated as additional evaluation metrics, with the values being 22.50, 1274.62 and 35.70, respectively. The training, evaluation, hyperparameter tuning and serialization of a random forest regressor model are successfully executed.

## 6. Results and Discussion

In this section, we present the results of each of the multimodal models along with a discussion, using mathematical preliminaries and the method employed in the validation steps.

### 6.1. Categorization and Classification of Tropical Cyclones

In summary, the results obtained after the ConvLSTM modeling and representation are essential in deciphering the potential of ConvLSTM in dealing with image sequences. The framework of subplots efficiently communicates the progression of frames in every series, distinguishing the primary path from further predictions. Table 4 provides the loss value, MAE and RMSE.

**Figure 10:**
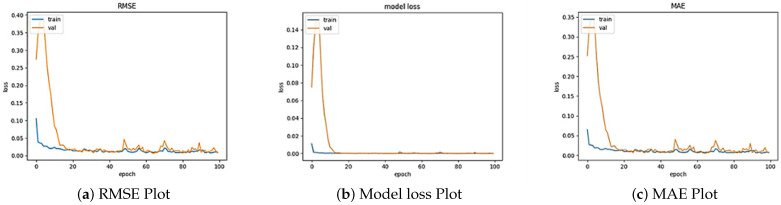
Plots for the train and validation data over 100 epochs.

The results obtained from the ConvLSTM modeling indicate strong performance in handling image sequences, with the performance metrics showing a test RMSE of 3.7870, a test loss of 14.3415 and a test MAE of 0.2858, reflecting the model’s efficiency in accurately predicting the outcomes. The visualization of the RMSE, model loss and MAE across the training epochs is shown in Figure 10. The use of Equations (2) and (4) reveals a consistent decrease in loss for both the training and validation sets, suggesting the model’s ability to effectively learn from the data and generalize to unseen instances. This highlights ConvLSTM’s suitability for the modeling of temporal dependencies inherent in image sequences, making it a powerful tool for tasks involving spatiotemporal relationships. The findings also indicate that the further tuning of the model could enhance its performance, encouraging the exploration of larger datasets and alternative architectures. Additionally, benchmarking ConvLSTM against other state-of-the-art models could provide insights into its relative strengths and weaknesses, ultimately affirming its potential applications in fields like meteorology and environmental monitoring.

**Figure 11:**
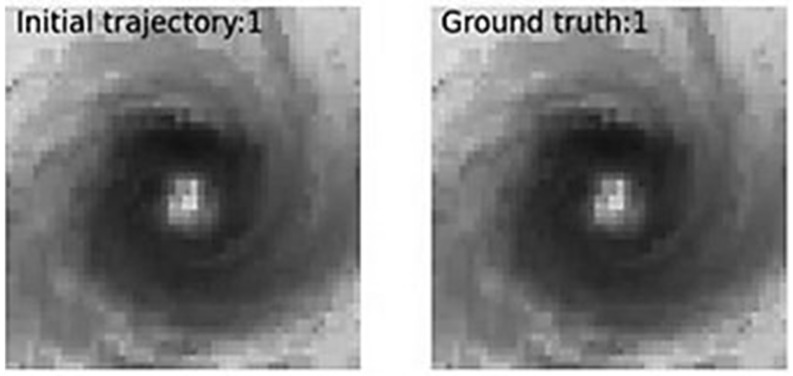
Initial trajectory predicted by the CNN compared with Ground truth.

The CNN model used here is known as a sequential model. Layers are stacked one at a time, and each layer has a different role. Convolutional layers help the model to understand patterns in the data. Pooling layers help in prioritization by focusing on the most important parts of the data. The flatten layer renders the data easy to work with by compressing information from previous layers. Dense layers analyze the data and make decisions. Figure 11 depicts the initial trajectory and ground truth of a TC. The initial trajectory is the predicted path of a TC, whereas the ground truth is the actual path.

This model is set to 100 epochs and a batch size of 60 images. Once the training is completed, metrics for each epoch are generated. The performance metrics of the model over each fold are tabulated in Table 5. To further evaluate the model’s performance, it was tested on four recent cyclones: Biparjoy (6 June 2023–19 June 2023), Mandous (6 December 2022–10 December 2022), Sitrang (22 October 2022–25 October 2022), Asani (7 May 2022–12 May 2022) and Gulaab (24 September 2021–28 September 2021). The model was tested on 10 images of each cyclone, and the results showed that the model performed well, correctly classifying 46 out of 50 images, as shown in Table 6.

The implementation of Real-ESRGAN provides valuable information for image enhancement. An environment has been successfully created to enable better-quality images to be produced by means of methodically determining the settings and applying Real-ESRGAN with certain key parameters.

**Figure 12:**
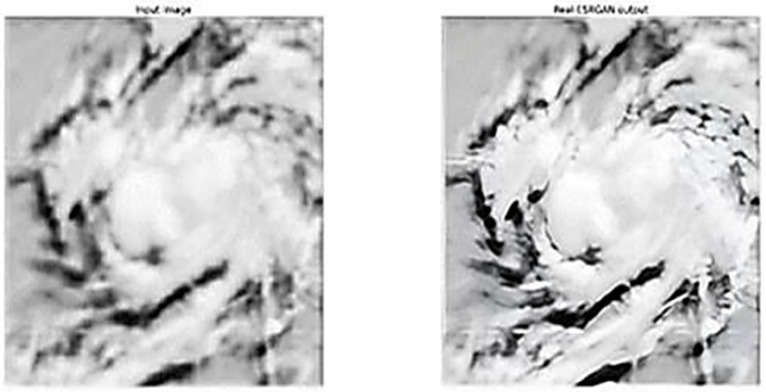
Satellite image before and after being fed to the GAN model.

At the core of our findings are the visual contrasts between the original input images and the respective Real-ESRGAN-enhanced ones. Such an approach gives a new dimension to evaluations based on subjective impressions. The results highlight the practical success of Real-ESRGAN in improving the image clarity by four times. The visual comparison is presented in Figure 12. The practicality of this method is also enhanced by its user-friendly interface, as well as the clear implementation process carried out. This demonstrates the capabilities of Real-ESRGAN and paves the way for further research in the domain of image improvement.

### 6.2. Prediction of Air Quality Index

The AQI research provides vital results and conclusions regarding its relevance in the context of environmental surveying. Using a more complex regression model for the prediction of the AQI shows that a large amount of effort is required in capturing all subtle factors associated with the data from the air before and after the occurrence of a TC, as provided by Equations (2), (4) and (5). This implies that the model’s reliability is based on certain environmental variables and on identifying and specifying the input features that are important for AQI prediction. This information is essential in analyzing the variables that affect the air quality and, consequently, the credibility of the model’s outcomes. The results regarding the performance metrics of the different models are tabulated in Table 7.

**Figure 13:**
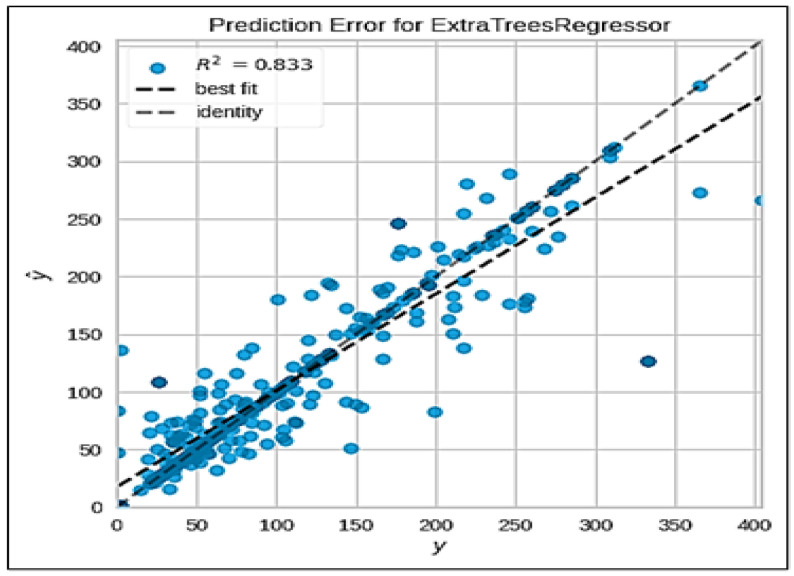
Prediction error for extra trees regressor.

Based on the previous comparisons, the best model is created. In this case, it specifies the extra trees model (‘et’) as the best model. Then, a plot is generated illustrating the feature importance for the best model, as visualized in Figure 13. This helps us to understand which features contribute the most to the model’s predictions. The best model is further tuned using the tune_model function, as shown in Table 8. It performs hyperparameter tuning to optimize the model’s performance. It is found that the root mean squared error is the metric to optimize. The training data are divided into 10 folds. In each iteration of the training process, the model is trained on nine of these folds and tested on the remaining fold. This process is repeated 10 times, each time using a different fold as the test set. The hyperparameter tuning process involves exploring a set of 10 different combinations of hyperparameter values. Since there are 10 folds for each of the 10 candidates, the total number of model fits or training cycles is 100 (10 folds × 10 candidates).

A plot displaying the error metrics is generated for the tuned model, as shown in Figure 13. It provides insights into the performance of the model. It can be inferred that the model performs well, having an $R^{2}$ value of 0.833. Finally, the tuned model is saved for future use. This system systematically investigates the relationships between tropical cyclones and the air quality by integrating TC-related meteorological variables into machine learning analysis. Through feature importance analysis, hyperparameter tuning and evaluation metrics, the system provides a comprehensive understanding of how TCs influence the air quality, contributing valuable insights for predictive modeling in areas prone to these extreme weather events.

Based on the observations from Table 9, the proposed method, utilizing a CNN for tropical cyclone (TC) categorization and the extra trees regressor (ETR) for AQI prediction, achieves accuracy of 92.02% for the CNN and an $R^{2}$ of 83.33% for the ETR. This approach stands out for its strong performance in both cyclone classification and air quality forecasting. It provides a highly accurate and reliable solution, surpassing or matching other existing methods, making it an optimal choice in predicting the AQI and categorizing tropical cyclones.

## 7. Conclusions and Future Work

This study presents a comprehensive framework for the processing of image sequences, seeking to enhance the prediction of the wind intensity and air quality index (AQI). It integrates several components, including a CNN for wind speed prediction, an AQI regression model, Real-ESRGAN for image enhancement and a Convolutional LSTM model, forming an intelligent system capable of handling visual data, spatiotemporal analysis and meteorological insights. The AQI regression model captures air quality fluctuations, while the CNN model provides accurate wind speed forecasts. The proposed multimodal model achieves accuracy of 92.02% for the CNN and an $R^{2}$ of 83.33% for the ETR.

This interdisciplinary approach should be able to support public health initiatives, environmental planning and disaster management. Additionally, this research underscores the role of cyclones in altering the environmental conditions, particularly by focusing on factors that improve the visibility after thunderstorms. Nevertheless, effective air quality management in both urban and rural areas post-storm requires context-specific approaches, given the complex relationship between visibility, weather events and PM2.5 levels. Another factor is to include pollution data along with satellite and meteorological data. This will improve the prediction of the AQI and aid in achieving the desired purpose.

## Figures and Tables

**Table 3:** Meteorological variables.

Variable	Unit	Description
T	°C	Average temperature
TM	°C	Maximum temperature
Tm	°C	Minimum temperature
SLP	hPa	Average atmospheric pressure at sea level
H	%	Average relative humidity
VV	Km	Average visibility
V	km/h	Average wind speed
VM	km/h	Maximum sustained wind speed

**Table 4:** Performance metrics of VonvLSTM.

Test RMSE	Test Loss	Test MAE
3.7870	14.3415	0.2858

**Table 5:** Performance under each fold.

Validation Fold	Fold 5	Fold 4	Fold 3	Fold 2
RMSE	12.03	11.69	11.59	10.97
MAE	8.523	8.62	8.7	9.85

**Table 6:** TC classification model performance.

Name of Cyclone	Total Images Batch Size	Correctly Classified	Incorrectly Classified
Biparjoy	10	9	1
Mandous	10	8	2
Sitrang	10	10	0
Asani	10	10	0
Gulaab	10	9	1

**Table 7:** Comparison of performance metrics for various regression models.

Model	Description	MAE	MSE	RMSE	$R^{2}$	RMSLE	MAPE	TT (S)
et	Extra Trees Regressor	19.4739	1269.152	35.3290	0.8159	0.7042	0.4351	0.347
rf	Random Forest Regressor	24.7298	1464.649	38.0743	0.7879	0.7978	0.4962	0.676
xgboost	Extreme Gradient Boosting	21.7267	1496.291	38.3864	0.7832	0.7335	0.4013	0.121
lightgbm	Light Gradient Boosting Machine	26.2971	1584.995	39.6437	0.7692	0.8116	0.4783	0.125
gbr	Gradient Boosting Regressor	31.7947	1940.334	43.9074	0.7201	0.9038	0.7111	0.138
dt	Decision Tree Regressor	27.4253	2583.185	49.9351	0.6299	1.0207	0.5258	0.026
ada	AdaBoost Regressor	42.5555	2793.173	52.8109	0.5915	1.0330	1.1294	0.115
knn	K-Nearest Neighbors Regressor	41.1119	3561.873	59.3394	0.4847	0.9044	0.8411	0.030
llar	Lasso Least Angle Regression	44.5045	3604.613	59.9010	0.4803	1.0608	1.1860	0.025

**Table 8:** Performance metrics for the best model after further tuning.

Fold	MAE	MSE	RMSE	$R^{2}$	RMSLE	MAPE
0	30.7998	1854.7757	43.0671	0.6931	0.8205	0.8340
1	35.8808	2303.8465	47.9984	0.6726	0.8423	0.8534
2	34.3332	2811.8899	53.0273	0.6932	0.9706	0.2943
3	37.6362	2645.2049	51.4316	0.6412	0.9215	1.3292
4	33.4335	2295.2797	47.9091	0.6379	0.6621	0.5222
5	35.1973	2012.3760	44.8595	0.7281	1.0181	0.3853
6	30.7601	1717.5258	41.4430	0.7026	0.9177	0.3959
7	33.8250	2171.7405	46.6019	0.7223	0.8538	0.3363
8	29.6900	1549.2964	39.3611	0.7076	1.1210	1.7976
9	34.2521	2430.1058	49.2961	0.6779	1.0744	1.4268
Mean	33.5808	2179.2041	46.4995	0.6876	0.9202	0.8175
Std	2.3723	381.1142	4.1231	0.0291	0.1278	0.5052

**Table 9:** Comparison of methods and accuracy across studies.

Work By	Methods Used	Accuracy
Jianyin Zhou et al. (2020) [1]	GC-LSTM Deep Learning Model	95.12% (Typhoon and Super Typhoon), 83.36% (Tropical Depression), 88.21% Average Accuracy
Chong Wang et al. (2020) [10]	CNN Model for Tropical Cyclone Track Prediction	Mean Absolute Error 27.8°
Jena et al. (2021) [8]	DCNN-Classifying Cloud Satellite Images	94% (images stored in the cloud are classified)
Nair et al. (2022) [7]	R-CNN for Mask Region Detection; CNN for Classifiers	86.55%
Shaohui Jin et al. (2022) [3]	Template Matching and Particle Swarm Optimization (PSOA)	High Accuracy for Center Location (comparison with NOAA Best Track)
Lam et al. (2023) [2]	YOLOv3 Deep Learning Model	78.31% (IoU = 0.1), 31.05% (IoU = 0.5)
P. Dong et al. (2023) [16]	850 hPa Relative Vorticity Cyclone Tracking Algorithm	Air pollution prediction: NC days: 36.5% frequency
		PM2.5 pollution: 27.4% frequency
		PM10 pollution: 16.3% frequency
Proposed Method	CNN for Caetogorization of TC, Extra Trees Regressor (ETR) for AQI	CNN: 92.02%, ETR: 83.33% ($R^{2}$)

## Data Availability

Data are contained within the article.
